# Clinical study of reoperation for acute type A aortic dissection

**DOI:** 10.3389/fcvm.2024.1340687

**Published:** 2024-03-01

**Authors:** Yi Feng, Xian-Tao Ma, Xiao-Xue Zhang, Akilu Wajeehullahi, Zi-Jun Chen, Shi-Liang Li, Cai Cheng

**Affiliations:** ^1^ Division of Cardiothoracic and Vascular Surgery, Tongji Hospital, Tongji Medical College, Huazhong University of Science and Technology, Wuhan, China; ^2^ Department of Cardiothoracic Surgery, Taikang Tongji (Wuhan) Hospital, Wuhan, China

**Keywords:** type A aortic dissection, reoperation, endovascular aortic repair, thoracoabdominal aortic replacement, clinical effect

## Abstract

**Objective:**

The initial operation for type A aortic dissection has limitations, and there may be a need for reoperation in cases such as giant pseudoaneurysm formation and reduced blood supply to the distal vessels. In this study, we collected case data of patients who underwent cardiac major vascular surgery at our hospital to analyze the effectiveness of reoperation treatment options for type A aortic dissection and to summarize our treatment experience.

**Method:**

Between June 2018 and December 2022, 62 patients with type A aortic dissection (TAAD) underwent reoperation after previous surgical treatment. Of these, 49 patients (45 males) underwent endovascular aortic repair (EVAR) with a mean age of (49.69 ± 10.21) years (30–75 years), and 13 patients (11 males) underwent thoracoabdominal aortic replacement (TAAR) with a mean age of (41.00 ± 11.18) years (23–66 years). In this study, we retrospectively analyzed the recorded data of 62 patients. In addition, we summarized and analyzed their Computed Tomographic Angiography (CTA) results and perioperative complications.

**Outcome:**

In the EVAR group, 47 patients (95.92%) were successfully implanted with overlapping stents, and 2 patients died in the perioperative period. Postoperative complications included cerebral infarction (4.08%), acute renal insufficiency (30.61%), pulmonary insufficiency and need for ventilator (6.12%), poor wound healing (2.04%), postoperative reoperation (16.33%), and lower limb ischemia (2.04%). In the TAAR group, 12 patients (92.31%) were successfully revascularized and 1 patient died in the perioperative period. Postoperative complications included cerebral infarction (7.69%), acute kidney injury (46.15%), pulmonary insufficiency and need for ventilator (15.38%), poor wound healing (30.77%) and postoperative reoperation (15.38%).

**Conclusion:**

According to the results of the study, compared with TAAR, EVAR was less invasive, faster recovery, and offered a better choice for some high-risk and high-age patients with comorbid underlying diseases. However, the rate of revascularization was higher after EVAR than TAAR due to vascular lesions. Compared with the use of ascending aortic replacement + hemi-aortic arch replacement for acute type A aortic dissection in many countries and regions, the use of ascending aortic replacement + aortic arch replacement + elephant trunk stent is more traumatic in China, but facilitates reoperation. For young patients, the choice of treatment should be individualized combining vascular lesions and long-term quality of life.

## Introduction

Aortic dissection (AD) is a fatal disease. The Stanford classification of aortic dissection was developed by Stanford University in 1970, which remains in place today (1). Acute type A aortic dissection (aTAAD) is considered to require immediate surgery with the primary aim of preventing aortic rupture, cardiac compression and partial restoration of branch vessel blood flow (2). And an increasing number of studies are advocating for a more aggressive surgical approach that extends the repair to the aortic root and aortic arch (3). In many countries and regions, surgical repair is mostly done by replacing the ascending aorta with a partial replacement of the aortic arch (4, 5). However, in China, a four-branch artificial vessel combined with an intraoperative stent system is mainly used to repair the ascending aorta, aortic arch, and part of the descending aorta (6). This type of surgery extends the scope of surgical repair of the lesion and avoids some of the complications of interventional therapy (7). Andreas Zierer with colleagues (8) found that about 13% of patients with aTAAD develop persistent dilatation of the thoracic aorta and about 8% develop persistent dilatation of the abdominal aorta after the intervention. Therefore, some patients with aTAAD, especially the young, require secondary surgical intervention to pursue long-term quality of life and life expectancy. Surgical treatment of the distal aortic lesions is thoracoabdominal aortic artery replacement (TAAR), endovascular aortic repair (EVAR), and aortic vascular bypass grafting combined with interventional stenting (known as a “hybrid” procedure) (9). TAAR used to be considered the gold standard for the treatment of thoracoabdominal aortic aneurysm/dissection, which could completely eradicate the distal diseased vessel, but TAAR is difficult and invasive, with many postoperative complications (10), and some patients cannot afford to undergo this operation. Since 1994, when Dake with colleagues first reported the successful treatment of aortic aneurysm by stent graft (11), with the rapid development of EVAR, this technique has been progressively applied to various types of aortic diseases. It was shown that the rates of complications such as spinal cord ischemia, renal injury and death after EVAR of abdominal aortic aneurysms were lower than those of TAAR (12). However, due to the varied vascular anatomy, many stents cannot be successfully implanted (13), and further evidence of long-term outcomes is needed. The reoperation option of TAAR or EVAR in patients with aTAAD should be carefully considered in the background of patient symptoms, CTA performance, and long-term patient beneficiation. In this study, we report the perioperative data of patients who underwent secondary surgery for aTAAD from June 2018 to December 2022, in order to explore the experience of reoperation for aTAAD.

## Material and methods

This study as retrospective research collects case data of aTAAD patients who underwent secondary surgery Division of Cardiothoracic and Vascular Surgery, Tongji Hospital, Tongji Medical College, Huazhong University of Science and Technology from June 2018 to December 2022 (Table 1). This study was approved by the Ethics Committee of our hospital (batch number:TJ-IRB20231107). We collected patients' perioperative information through the centre's electronic medical records database. During this period, 62 patients underwent reoperation after previous surgical treatment for Stanford type A aortic dissection. Of these patients, 20 patients underwent ascending aortic repair, 3 patients underwent ascending aortic + partial aortic arch repair, and 41 patients underwent ascending aortic + total arch repair using an elephant trunk stent (28 × 100 mm CRONUS, Shanghai MicroPort Endovascular MedTech, CN). In all 62 patients, 49 underwent EVAR (Thirty-five cases of thoracic endovascular aortic repair, five cases of abdominal endovascular aortic repair, eight cases of thoracoabdominal endovascular aortic repair, and one case of arch endovascular aortic repair) and 13 patients underwent TAAR.

**Table 1:** Baseline information.

	Group. EVAR (*n* = 49)	Group. TAAR (*n* = 13)	*P-*value
Age/years (range)	49.69 ± 10.21 (30–75)	41.00 ± 11.18 (23–66)	0.017
Male sex	45 (91.84%)	11 (84.62%)	0.799
Hypertension	41 (83.67%)	5 (38.46)	0.003
Chronic obstructive pulmonary disease	1 (2.04%)	–	0.790
Chronic renal failure	2 (4.08%)	–	0.622
Cerebrovascular disease	2 (4.08%)	–	0.622
Chronic stomach disease	2 (4.08%)	–	0.622
Coronary artery occlusive disease	5 (10.20%)	–	0.530
History of smoking	14 (28.57%)	2 (15.38%)	0.542
History of drinking	9 (18.37%)	1 (7.69%)	0.613
The method of the first surgery			
Ascending aortic repair	16 (32.65%)	4 (30.77%)	0.838
Ascending aortic + partial arch repair	3 (6.12%)	–	0.487
Ascending aortic + total arch repair	32 (65.31%)	9(69.23%)	0.790

We analyzed the results and compared these results.

### EVAR group

Indications for this surgery are:

1. Severe vascular lesions resulting in a small true lumen
2. Patients with obvious symptoms such as chest and abdominal pain and lower extremity weakness.
3. Newly developed dissection leading to circulatory instability.
4. The breach of the dissection avoids the visceral vascularization area.
5. Rule out Marfan syndrome and connective tissue disease.

**Figure 1:**
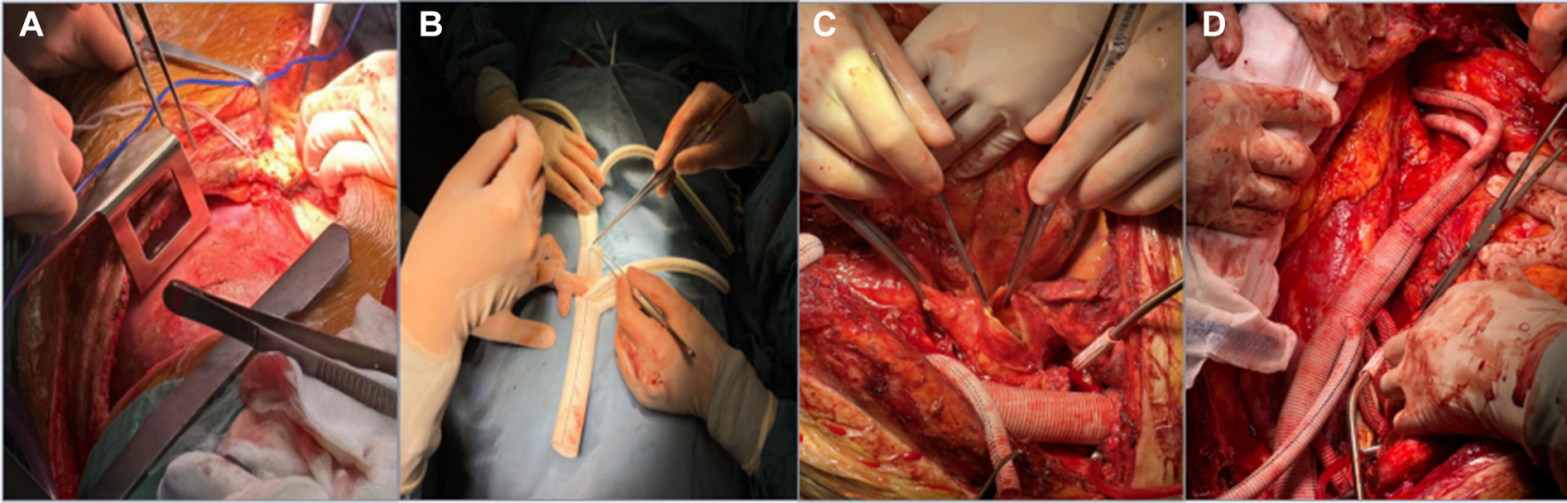
The process of TAAR. (**A**) A joint left thoracoabdominal incision; (**B**) anastomosis of a four-branch artificial vessel to a *Y*-shaped artificial vessel; (**C**) after iliac artery revascularization, the aneurysm body is incised to look for visceral vascular openings; (**D**) thoracoabdominal aortic revascularization complete.

Patients were placed in the supine position with general or local anesthesia, and an inguinal incision was taken to isolate the femoral artery for backup. Heparinized, the femoral sheath (6F, Demax Medical, CN) was punctured and placed, and a guidewire (0.035"-150 cm Blackeel, APT MEDICAL, CN) guided the contrast catheter (5F 0.035"-100 cm PIG-CSC-20. COOK Medical, USA) into the true luminal aortic arch for imaging. The post-aTAAD aortic morphology, dissection, and pseudoaneurysm can be clarified by contrast. A rigid guidewire (0.035"-260 cm susrail, APT Medical, CN) was exchanged and a stent delivery system* was implanted along the rigid guidewire via the femoral artery, and the overlapping stent was released at a predetermined location under radiation (Figure 1). The femoral artery is sutured, hemostatic, and the incision is closed layer by layer. Thirty-five cases (71.43%) of thoracic endovascular aortic repair (TEVAR) from the beginning of the thoracic aorta, five cases (10.20%) of abdominal endovascular aortic repair (AEVAR) from renal artery level to iliac artery bifurcation level, eight cases (16.33%) of thoracoabdominal endovascular aortic repair, and one case (2.04%) of arch endovascular aortic repair by fenestrate technique. (*Note: Stent delivery system Medtronic. USA, Lifetech.CN, MicroPort.CN).

### TAAR group

Indications for this surgery are:

1. Severe vascular lesions resulting in a small true lumen.
2. Patients with obvious symptoms such as chest and abdominal pain and lower extremity weakness.
3. Newly developed dissection leading to circulatory instability.
4. The aortic arch diameter is >5.0 cm (or >4.5 cm in combination with Marfan's syndrome or aortic arch rupture) or when the aortic arch dilates at a rate of >0.5 cm/year.

**Figure 2:**
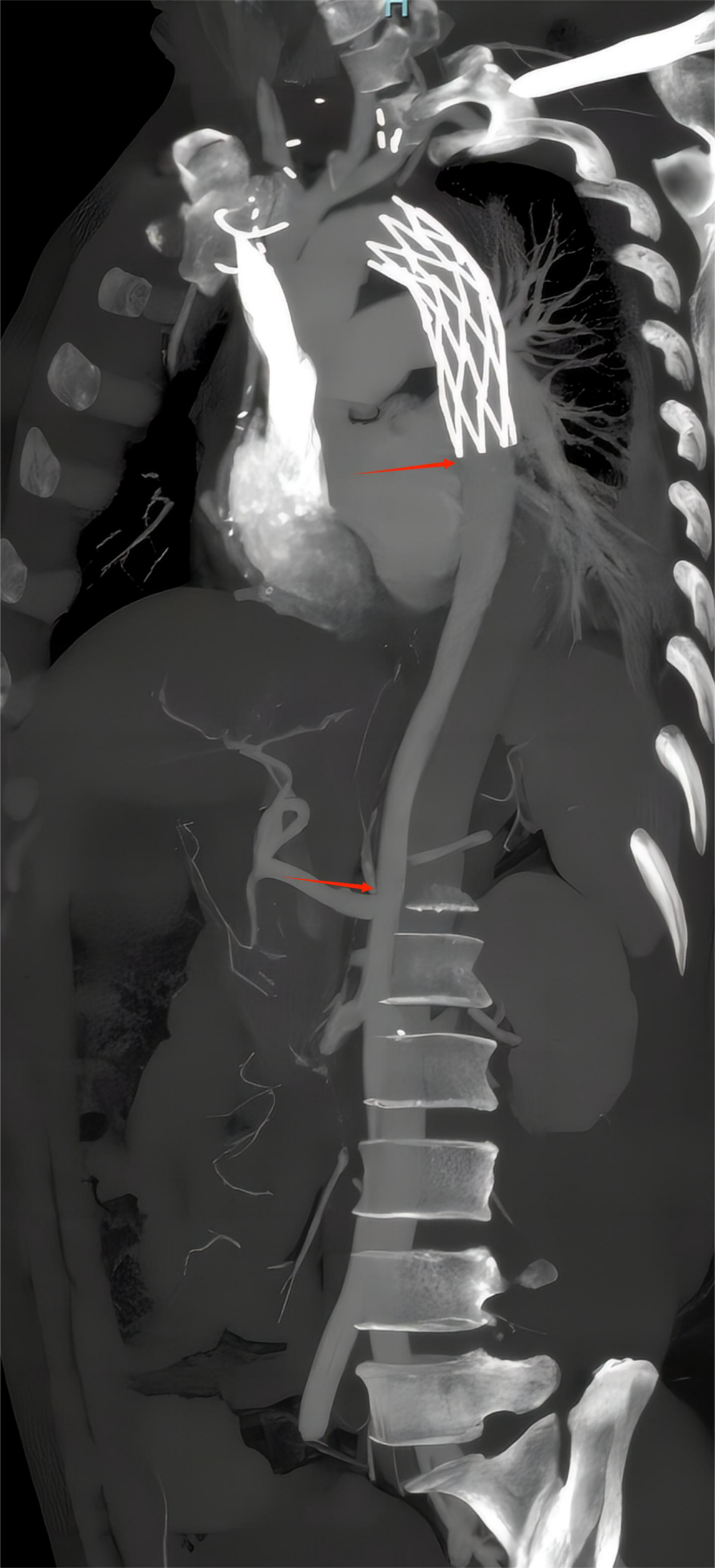
Zoning of CTA. The s1 plane and s2 plane in the longitudinal section.

Preoperative preparation Nasal feeding tube placement, gastrointestinal decompression. The anesthesiologist administered general anesthesia and performed double-lumen tracheal intubation. The patient was placed in the right lateral position, and a joint left thoracoabdominal incision was made starting from the midpoint of the medial border of the scapula and the spinous process, down along the external border of the rectus abdominis muscle to above the pubic symphysis. The thorax was entered through the left 4th–7th intercostal space, the rib arch was cut, and the thoracic descending aorta was freed, as also the distal abdominal aorta and bilateral common iliac arteries were freed behind the peritoneum. After whole-body heparinization, four branches of the artificial vessels were taken for vascular replacement. (In some cases, a four-branch artificial vessel combined with a *Y*-shaped artificial vessel was used for revascularization). First, two 10 mm branches of the artificial vessel were anastomosed to the left and right common iliac arteries, respectively, and the other 8 mm branch was connected to a single pump of the extracorporeal circulation machine, and the other branch and the ends of the trunk were blocked to establish a transfusion channel. The thoracic descending aorta was directly blocked with two blocking clamps near the beginning of the left subclavian artery, and the trunk of the four-branch artificial vessel was anastomosed end-to-end with the beginning of the thoracic descending aorta, and the proximal blocking clamp was removed. The systemic blood supply was restored via the artificial vessels. The abdominal aorta was blocked above the abdominal trunk, the thoracic aortic aneurysm was dissected longitudinally, the T6-L1 intercostal artery bed was trimmed and preserved, the intercostal artery was reconstructed, and the spinal cord blood supply was restored. The celiac trunk, superior mesenteric artery and left and right renal artery openings were trimmed into island-shaped vascular pieces and anastomosed with the distal ends of the four branch vascular trunks to restore blood supply to the abdominal organs and ligate the branches for perfusion. The lumbar artery, sub mesenteric artery and other vessels were sutured at the opening of the abdominal aorta to stop bleeding (Figure 2). After completion of revascularization, heparin was neutralized and the thoracic and abdominal cavities were carefully examined for hemostasis. Retroperitoneal and thoracic drains were placed, and the incision was closed layer by layer.

### Outcomes

#### EVAR group

We mainly analyzed the postoperative complication rate and mortality in 49 patients with EVAR and used preoperative and postoperative CTA to evaluate surgical outcomes. In these patients, the mean operative time was (127 ± 62) min and the postoperative hospital stay was (9.3 ± 7.2) d. Of the 49 patients, 47 cases (95.92%) of EVAR were clinically successful. There were 2 perioperative deaths, including 1 postoperative complication of multiple organ dysfunction syndrome (MODS) which manifested as delirium, abnormal liver and kidney functions and 1 intraoperative vessel rupture. Other perioperative complications included postoperative cerebral infarction in 2 patients which manifested as hemiplegia, considered to be related to aortic false lumen thrombosis and stent migration. These 2 patients were treated with hyperbaric oxygen, anticoagulation, lipid-lowering, and functional exercises, and their limb function was significantly improved. Renal insufficiency due to acute kidney injury (AKI) in 15 patients, 4 of whom showed symptoms of oliguria, anuria and underwent continuous renal replacement therapy (CRRT). 3 patients had postoperative pulmonary insufficiency requiring non-invasive ventilation assistance, which was thought to be associated with history of previous lung disease and postoperative pulmonary atelectasis after general anesthesia. 8 patients underwent reoperation, 4 patients underwent EVAR for distal stenosis and inadequate perfusion, 1 patient underwent TAAR for persistent dilatation of distal entrapment aneurysm, 2 patients underwent sternal fixation for early poor sternal healing, 1 patient underwent debridement and suturing for poorly healing incision, and 1 patient presented with gangrene of the lower limbs in early stage with ineffective improvement of blood supply.

**Figure 3:**
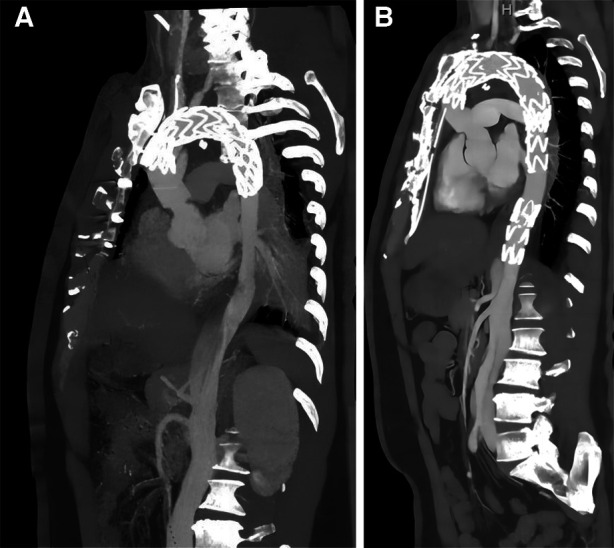
Comparison of EVAR before and after surgery by CTA. The second surgery underwent EVAR (a anteriorly and b posteriorly) with good results and significant improvement in true vena cava flow, but with residual abdominal aortic dissection, possibly facing reoperation.

**Figure 4:**
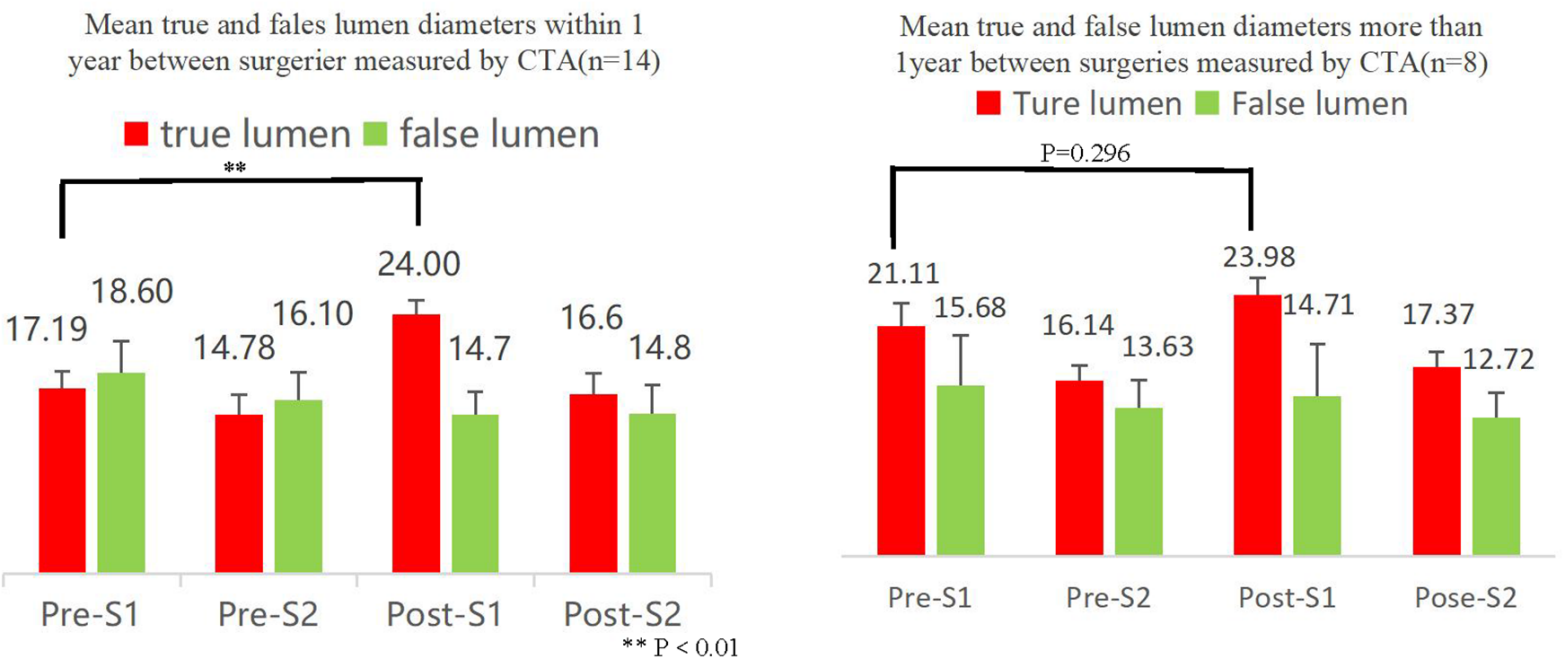
Lumen diameter as assessed by CTA (*n* = 22 out of 49 EVAR patients).

Outcomes of EVAR were assessed by preoperative and postoperative CTA images (Figure 3). A total of 22 patients underwent CTA both preoperatively and 3 months postoperatively (Table 2), and the remaining patients did not undergo CTA preoperatively and/or postoperatively. The end of the stent or the beginning segment of the thoracic aorta was selected as the first measured section (S1), and the level of the opening of the celiac trunk artery was the second section (S2), and the size of the true lumen (T) and false lumen (F) diameters were measured by CTA to assess the short-term treatment effect after EVAR surgery (Figure 4).

**Table 2:** Time to first surgery.

Operation time (from the first surgery)	Group. EVAR (*n* = 49)	Group. TAAR (*n* = 13)	*P-*value
Within 1 month	8 (16.33%)	–	0.273
Within 3 months	6 (12.24%)	2 (15.38%)	0.869
Within 6 months	8 (16.33%)	1 (7.69%)	0.731
Within 1 year	6 (12.24%)	–	0.424
More than 1 year	21 (42.86%)	10 (76.92%)	0.029

Patients who underwent EVAR within 1 year had significantly better postoperative true lumen improvement than patients who underwent EVAR after 1 year.

#### TAAR group

Thirteen patients underwent TAAR at the second surgery, they had mean operative time (522 ± 65) min, mean cardiopulmonary bypass time (153 ± 31) min, mean intraoperative transfusion of erythrocytes (6.3 ± 3.6) U, plasma (808 ± 483) ml, and mean hospital stay postoperatively (15.2 ± 6.2) days. Surgery was successful in 12 patients, but one patient died of intraoperative disseminated intravascular coagulation (DIC). Postoperative AKI occurred in 6 patients, which was related to intraoperative renal ischemia-reperfusion injury, but with the improvement of renal perfusion blood flow and the use of renal protective drugs, the renal function of these 6 patients gradually improved without the need for CRRT. 4 patients had poor incision healing, 2 patients had poor incision healing with infection and reopened for debridement and suturing, which was directly related to the long and traumatic surgical incision and poor postoperative incision care. 10 patients developed varying degrees of postoperative lung infections, which may be related to a history of previous lung disease, ventilator-associated pneumonia (VAP) due to high trauma of general anesthesia surgery, and aspiration pneumonia (HP) due to prolonged bed rest of the weakened patient. 11 patients developed postoperative pleural and/or ascites, which was related to diffuse exudation from a larger wound, early postoperative fasting and rehydration, and malnutrition. Most of the patients improved their lung infections with noninvasive ventilator-assisted ventilation and antibiotic therapy.

To make the results more accurate, we compared the complications of 2 different procedures performed in patients whose initial procedure was ascending aorta + total arch repair (Table 3).

**Table 3:** Postoperative complications.

Complications	Group. EVAR (*n* = 33)	Group. TAAR (*n* = 9)	*P-*value
Death or abandoning treatment	1 (3.03%)	1 (11.11%)	0.387
Cerebral infarction	1 (3.03%)	1 (11.11%)	0.387
Renal insufficiency	22 (66.67%)	5 (55.56%)	0.538
Pulmonary insufficiency and need for ventilator	1 (3.03%)	2 (22.22%)	0.111
Poor wound healing	1 (3.03%)	3 (33.33%)	0.026
Revascularization	3 (9.09%)	–	0.475

## Discussion

aTAAD is a life-threatening condition and advocates aggressive surgery to prevent death due to ruptured aortic dissection (14). However, postoperative outcomes of emergency surgery vary widely and may be closely related to the extent of the entrapment, poor perfusion syndrome, location of the primary entrance tear, haemodynamic instability, painless entrapment and delayed clinical diagnosis, age, other comorbidities, and general experience of the surgical team (15). In some centres, the perioperative mortality rate for aTAAD has been reduced to approximately 10%–15% with a 5-year survival rate of approximately 70%–90% (16, 17). However, even if aTAAD surgery is successful, postoperative vascular-related mortality and reoperation rates remain high (18).

With the development of anaesthesia, cardiopulmonary access techniques and strategies for organ perfusion protection, ascending aortic replacement combined with total arch replacement using a four-branched graft with stenting of the elephant trunk has become the standard procedure in a part of the national centres (19), and this type of procedure for the treatment of aTAAD has a perioperative mortality rate of approximately 6% and an incidence of reoperation of approximately 4% (20), which is lower than most of the data levels reported in the literature.Some studies have shown a low incidence of postoperative reintervention procedures after aTAAD (16% at 10 years) (21), and favorable long-term results with conventional open repair (22, 23). The treatment modalities for aTAAD reoperation include TAAR, EVAR and hybrid surgery. This study focuses on the TAAR and EVAR modalities. Preoperative CTA was performed to assess vascular lesions (some patients combined data from external hospital examinations and intraoperative digital subtraction angiography).

Successful endovascular repair is defined as sealing the main fissure without residual endoleak at the end of the procedure (24). Surgical success does not guarantee distal vascular disease, and patients may still face a third or even more aortic surgeries after endovascular repair. In some studies,reintervention rates are higher for endovascular repair than for open surgery (25). However, in this study, there was no statistical difference regarding the rate of revascularization after EVAR and TAAR procedures. This is closely related to the sample size and follow-up time. In fact, there were some patients who failed to improve the true lumen effectively as seen in the CTA results after EVAR surgery and predictably required reoperation. With longer follow-up, the revascularization rate after the two procedures may show a significant difference. The most common reasons for re-intervention are endoleak, false lumen perfusion and aortic dilatation as well as new entrapment (26). The feasibility of endovascular repair has been demonstrated in elderly, frail and high-risk patients who were previously considered unsuitable for open surgery (27–29). Early repair of aortic dissection lesions in the acute phase has been found to result in better vascular remodeling outcomes (30). EVAR treatment of patients in this study at 1 year was superior to patients after 1 year for true lumen improvement. Expansion of the true lumen after endovascular repair is associated with the selection of an oversizing stent and with vascular remodeling. And in our study, it was found that 40.09% (9/22) of the patients failed to have a significant true lumen condition after EVAR. The large diameter of the false lumen in this subset of patients may be a significant contributor to the poor postoperative results of EVAR. The ascending aortic replacement combined with aortic arch replacement and elephant trunk stent for aTAAD in China provides a good convenience for the second endovascular repair. Simplified surgical steps, avoiding complex operations such as bypass grafting or fenestrate technology (31). It is important to focus on the long-term prognosis of the patient rather than on new procedures when administering appropriate treatment (32).

The surgical treatment strategy of reoperation is closely related to the scope and degree of aortic disease treated by the first operation. The blood pressure and tension of distal aortic wall increased in patients with proximal aortic dissection for the first time, which increased the risk and difficulty of distal resurgical treatment (33). If the patient's condition permits, TAAR should be performed as much as possible, especially in patients with Marfan's syndrome, to avoid reoperation if possible and to simplify the operation when the next operation is required. More importantly, TAAR can also be performed when the aortic arch diameter is >5.0 cm (or >4.5 cm in combination with Marfan's syndrome or aortic arch rupture) or when the aortic arch dilates at a rate of >0.5 cm/year. In our group, EVAR was used in the management of distal lesions, and the follow-up results were good. Some studies have reported that EVAR can be used to treat distal lesions again with more satisfactory results soon (34), but the long-term clinical effect remains to be observed and more clinical studies are needed to confirm this. EVAR is not recommended for patients with Marfan's syndrome and other connective tissue pathologies, and studies have shown that 66%–83% of such patients require surgical intervention in the short term after endovascular intervention (35). It is evident that whenever possible proximal aortic entrapment should be avoided when dealing with the proximal regardless of the surgical procedure chosen, to avoid leaving behind a torn aortic wall, to prevent pseudoaneurysm formation, aneurysmal dilatation of the root, or even recurrent entrapment, and to reduce the probability of proximal reoperation.

The timing of reoperation should be selected as far as possible before emergency operation is needed. Statistical analysis shows that emergency hand surgery is a risk factor for death of reoperation, and the fatality rate of patients undergoing emergency hand operation is significantly higher than that of patients undergoing elective operation (36, 37). Therefore, it is even more important to develop a complete and close lifelong follow-up plan for patients after aTAAD, and to develop an elective surgical plan for patients who may undergo reoperation rather than being forced to undergo emergency surgical treatment again. Especially for patients with combined Marfan's syndrome, the probability of reoperation is higher because of the high incidence of new or aggravated lesions in the distal aorta after aortic root surgery (38). Of course, in the case of emergency situations, such as recurrent entrapment combined with hemothorax, poor perfusion of viscera and limbs, and uncontrollable hypertension (39), emergency surgery should be performed to remove and replace the diseased vessel in a timely manner.

## Conclusion

(1) The use of ascending aortic replacement + total arch replacement + elephant trunk stent implantation effectively reduces the difficulty of reoperation for the treatment of thoracoabdominal aortic vasculopathy.
(2) Early EVAR is more effective for vascular remodeling after surgical treatment of aTAAD by ascending aorta + total aortic arch repair.
(3) EVAR surgery is less invasive compared to TAAR surgery; however, in terms of long-term outcomes, EVAR surgery has a higher rate of revascularization than TAAR surgery. Therefore, the approach to revascularization in patients after aTAAD should be individualized to take into account the objective situation.

### Limitation

(1) Insufficient sample size.
(2) Insufficient follow-up time, long-term effects to be further observed.

## Data Availability

The original contributions presented in the study are included in the article/Supplementary Material, further inquiries can be directed to the corresponding authors.
